# Supplementary material for: Lactation-associated macrophages exist in murine mammary tissue and human milk
Source: Nat Immunol. 2023 Jun 19;24(7):1098–109. doi: 10.1038/s41590-023-01530-0 (PMC10307629; doi:10.1038/s41590-023-01530-0)
Supplement: Supplementary file 1 — Reporting Summary [file 41590_2023_1530_MOESM1_ESM.pdf]

Corresponding author(s): Melanie Greter

Last updated by author(s): May 8, 2023

Reporting Summary

Nature Research wishes to improve the reproducibility of the work that we publish. This form provides structure for consistency and transparency in reporting. For further information on Nature Research policies, see our Editorial Policies and the Editorial Policy Checklist.

Statistics

For all statistical analyses, confirm that the following items are present in the figure legend, table legend, main text, or Methods section.

n/a	Confirmed	
☐	☒	The exact sample size (*n*) for each experimental group/condition, given as a discrete number and unit of measurement
☐	☒	A statement on whether measurements were taken from distinct samples or whether the same sample was measured repeatedly
☐	☒	The statistical test(s) used AND whether they are one- or two-sided<br>*Only common tests should be described solely by name; describe more complex techniques in the Methods section.*
☒	☐	A description of all covariates tested
☐	☒	A description of any assumptions or corrections, such as tests of normality and adjustment for multiple comparisons
☐	☒	A full description of the statistical parameters including central tendency (e.g. means) or other basic estimates (e.g. regression coefficient) AND variation (e.g. standard deviation) or associated estimates of uncertainty (e.g. confidence intervals)
☐	☒	For null hypothesis testing, the test statistic (e.g. *F*, *t*, *r*) with confidence intervals, effect sizes, degrees of freedom and *P* value noted<br>*Give P values as exact values whenever suitable.*
☒	☐	For Bayesian analysis, information on the choice of priors and Markov chain Monte Carlo settings
☒	☐	For hierarchical and complex designs, identification of the appropriate level for tests and full reporting of outcomes
☒	☐	Estimates of effect sizes (e.g. Cohen's *d*, Pearson's *r*), indicating how they were calculated

Our web collection on statistics for biologists contains articles on many of the points above.

Software and code

Policy information about availability of computer code

Data collection	Flow cytometry data were collected using FACS Diva Software v9.1 or SpectroFlo® Software version 3.1.0.<br>Microscopy images were acquired using LAS X software.
Data analysis	FlowJo 10.6.2 and 10.8.1 (Tree star) was used for the Flow cytometry data analysis;<br>R and R studio v3.2-v4.0 was used for bioinformatics analysis;<br>UMAP and FlowSOM algorithms were used from publicly available sources, which are cited in the paper;<br>GraphPad Prism v 7.03 and 9.4.0 (Graphpad Software Inc.) was used to prepare graphs and perform statistical analysis;<br>LAS X (Leica) and Imaris (Bitplane) software were used for image analysis;<br>Seurat v4.1 in conjunction with Platypus v3.4.1 for scRNAseq general analysis, plots and Tabula Muris data preparation and annotation transfer;<br>Symphony v0.1.0 for integration with Tabula Muris;<br>Limma, edgeR and 3dVolcano for polar volcano.<br>The analysis code has been uploaded to https://github.com/gustaveroussy/FG-Lab.

For manuscripts utilizing custom algorithms or software that are central to the research but not yet described in published literature, software must be made available to editors and reviewers. We strongly encourage code deposition in a community repository (e.g. GitHub). See the Nature Research guidelines for submitting code & software for further information.

Data

Policy information about availability of data

All manuscripts must include a data availability statement. This statement should provide the following information, where applicable:

- Accession codes, unique identifiers, or web links for publicly available datasets
- A list of figures that have associated raw data
- A description of any restrictions on data availability

Publicly available datasets: GRCm38.p6 Release M23, GRCh38.p13, Ensembl database, MoMAC-VERSE.
The mouse mammary gland and human milk scRNA-seq datasets are deposited in the Genome Expression Omnibus under the accession numbers: GSE230697 and GSE230749, respectively. The mass spectrometry proteomics data have been deposited to the ProteomeXchange Consortium via the PRIDE partner repository with the dataset identifier PXD041711.

Field-specific reporting

Please select the one below that is the best fit for your research. If you are not sure, read the appropriate sections before making your selection.

☒ Life sciences ☐ Behavioural & social sciences ☐ Ecological, evolutionary & environmental sciences

For a reference copy of the document with all sections, see nature.com/documents/nr-reporting-summary-flat.pdf

Life sciences study design

All studies must disclose on these points even when the disclosure is negative.

Sample size	The sample size was chosen according to 3R principles, and normally included 3-5 mice per group, which is usually enough to get a statistical difference should there be any. No statistical method to predetermine sample size was used. Details on sample sizes are provided in figure legends and methods section.
Data exclusions	No animals were excluded from analysis unless re-genotyping revealed a wrongly determined genotype: then the animal was re-allocated in a correct group. FACS data were pregated for certain markers as described in figure legends. For scRNAseq data analysis, low quality cells exclusion and doublets filtering was done as described in methods section.
Replication	All experiments were independently repeated at least once and the number of experiments was stated in the figure legends. Single cell RNA sequencing analysis was performed once.
Randomization	No special method of randomization was used. The mice were randomly allocated to cages upon arrival from vendour or weaning. Covariates influence was controlled by keeping mice in the same conditions and repeating experiments several times.<br>Experimental and control groups were distributed to equal numbers.
Blinding	It was not possible to make the investigators completely blinded to group allocation, since usually the same experimenter performed genotyping, experimental procedures, tissue harvest and data analysis, however, whenever possible, experimenters tried not to pay attention to experimental group identity before the final steps of data analysis.

Reporting for specific materials, systems and methods

We require information from authors about some types of materials, experimental systems and methods used in many studies. Here, indicate whether each material, system or method listed is relevant to your study. If you are not sure if a list item applies to your research, read the appropriate section before selecting a response.

Materials & experimental systems

n/a	Involved in the study	
☐	☒	Antibodies
☒	☐	Eukaryotic cell lines
☒	☐	Palaeontology and archaeology
☐	☒	Animals and other organisms
☐	☒	Human research participants
☒	☐	Clinical data
☒	☐	Dual use research of concern

Methods

n/a	Involved in the study	
☒	☐	ChIP-seq
☐	☒	Flow cytometry
☒	☐	MRI-based neuroimaging

Antibodies

Antibodies used	For flow cytometry on murine cells, the following antibodies were used: I-A/I-E (BioLegend art. 107620 and BD art. 746197, clone M5/114.15.2), CD11b (BD art. 612800 clone M1/70), CD11c (eBioscience art. 35-0114-82 and BioLegend art. 117331, clone N418),

CD45 (BD art. 564279 clone 30-F11), Ly6C (BioLegend art. 128037 and art. 128023 clone HK1.4 and BD art. 553104 clone AL-21), Ly6G (BD art. 565707 clone 1A8), Siglec-F (BD art. 5626817 clone E50-2440), CD3 (Biolegend art. 100236 clone 17A2), NK1.1 (Biolegend art. 108745 clone PK136), CD19 (BD art. 612971 clone 1D3), CD64 (Biolegend art. 139309 clone X54-5/7.1), F4/80 (Biolegend art. 123112, art. 123135 clone BM8), CD169 (eBioscience art. 12-5755-80 clone SER-4), MerTK (eBioscience art. 25-5751-82 clone DS5MMER), CD206 (Biolegend art. 141734 clone C068C2), Lyve1 (eBioscience art. 50-0443-82 clone ALY7), CD38 (Biolegend art. 102714 clone 90), CX3CR1 (Biolegend art. 149027 and art. 149029 clone SA011F11), Dectin-1 (Biolegend art. 144303 clone RH1 and Biorad art. MCA2289A647T clone 2A11), XCR1 (BioLegend art. 148220 clone ZET), TNF (Biolegend art. 506328 clone MP6-XT22), and proIL-1b (eBioscience art. 25-7114-80 clone NJTEN3). Prior to detailed analysis cells were always gated on single and live cells. Dead cells were excluded with the Zombie NIR Fixable Viability Kit (Biolegend art. 423106). Click-iT EdU Cell Proliferation Kit (ThermoFisher) was used for the cell proliferation assay.
For flow cytometry on human cells CD11b (Biolegend art. 301324 clone ICRF44), CD11c (ThermoScientific art. MHCD11c18 clone BU15), CD14 (BD art. 741441 clone M5E2), CD16 (Biolegend art. 302020 clone 3G8), CD64 (Biolegend art. 305005 clone 10.1), CD66b (BD customized clone G10F5), CD3 (BD art. 612895 clone UCHT1), CD4 (BD art. 300508 clone RPAT4), CD8 (Life Technologies art. MHCD0829 clone 3B5), Siglec8 (Biolgened art. 347109 clone 7C9), MerTK (Biolegend art. 367609 clone 590H11G1E3), FceR1 (BD art. 747782 clone CRA1), CD163 (Biolegend art. 333629 clone GHI/61), CX3CR1 (BD art. 746723 clone 2A91), HLA-DR (BD art. 565073 clone G46-6), CD56 (BD art. 612766 clone NCAM16.2).

For histology, the following antibodies were used: anti-smooth muscle actin (Dako art. M0851 clone 1A4), anti-CD11c conjugated to AlexaFluor 594 (BioLegend art. 117346 clone N418), anti-Lyve1 conjugated to eFluor 660 (eBioscience art. 50-0443-82 clone ALY7), anti-Ki67 conjugated to PE-Cy7 (eBioscience art. 25-5698-82 clone SOLA15), anti-Iba1 (Wako art. 011-27991), anti-GFP (Nacalai Tesque art. 04404-84), anti-SMA (Sigma art. F3777 clone 1A4), anti-CD64 (Bio-Rad art. MCA5997 clone AT152-9), anti-Dectin-1 (Bio-Rad), anti-CD138 (BioLegend clone 281-2), anti-IgA (Southern Biotech art. 1040-01), anti-MHCII (BioLegend art. 107610 clone M5/114.15.2 (M5/114).

Anti-CSF-1 neutralizing antibody (clone 5A1) and isotype control antibody (rat IgG1, clone HPRN) were purchased from Bio X Cell (West Lebanon, NH, USA) and were administered i.p. at a dose of 0.2-0.3 mg.

Validation	All antibodies used in this study have been previously validated by their manifacturers (the details can be checked on the manufacturers' websites by catalogue number), and further titrated and compared with FMO (FACS antibodies) in our lab.

Animals and other organisms

Policy information about studies involving animals; ARRIVE guidelines recommended for reporting animal research

Laboratory animals	Female mice were normally used at the age of 7-12 weeks. The following strains were used: C57BL/6Rj, Csf1rfl/fl, ItgaxCre, Ms4a3Cre, Ccr2CreER-mKate, Il34LacZ, Ccr2-/-, Cx3cr1GFP, R26Ai14.
Wild animals	No wild animals were used in the study.
Field-collected samples	No field-collected samples were used in the study.
Ethics oversight	All experimental procedures at the University of Zurich were performed in accordance with Swiss Federal regulations and approved by the Cantonal Veterinary Office of Zurich.

Note that full information on the approval of the study protocol must also be provided in the manuscript.

Human research participants

Policy information about studies involving human research participants

Population characteristics	Human milk samples were collected at the University Hospital Zurich from healthy women, aged 20 to 40 years [mean (SD) 32.0 (5.0)], within 4 to 63 days after delivery.
Recruitment	The recruitment of the study participants and the collection of human milk samples took place at the Department of Neonatology of the University Hospital Zurich. Women eligible as study participants were contacted by the project leader, informed about the present study protocol and asked for consent for participating to the study. The informed consent process included ample time for consideration given to the participants and opportunity to ask questions. No compensation or payments were given to the study participants. There were no selection biases. The milk donors could withdraw their consent at any point in time without justification.
Ethics oversight	The Federal Act on Research involving Human Beings (HRA, RS 810.30) and the Ordinance on Human research with the exception of clinical trials (HRO, RS 810.301, Art. 6-23). The study protocol was approved by the Swiss Ethics Commission of the Canton of Zurich (BASEC-Nr. 2020-00542) and all the subjects participating to the study signed an inform consent before the enrollment.

Note that full information on the approval of the study protocol must also be provided in the manuscript.

Flow Cytometry

Plots

Confirm that:

☒ The axis labels state the marker and fluorochrome used (e.g. CD4-FITC).

☒ The axis scales are clearly visible. Include numbers along axes only for bottom left plot of group (a 'group' is an analysis of identical markers).

☒ All plots are contour plots with outliers or pseudocolor plots.

☒ A numerical value for number of cells or percentage (with statistics) is provided.

Methodology

Sample preparation	Mice were sacrificed by CO2 inhalation and intracardially perfused with phosphate buffered saline (PBS; pH 7.4, Gibco). After perfusion, left and right abdominal mammary glands were dissected, and inguinal lymph nodes removed. All the samples were cut into small pieces in an Eppendorf tube, followed by digestion in 0.4 mg/ml collagenase type IV (Worthington) and 0.04 mg/ml DNase I in Ca2+/Mg2+ HBSS supplemented with 10% FCS for 40 mins at 37°C, while shaking. Samples were homogenized with a 18 G needle and a syringe and filtered through a 100 µm cell strainer to obtain a homogeneous cell suspension. Cells were once washed in PBS, resuspended in red blood cell lysis (0.16 M NH4Cl, 0.11 M KHCO3, 0.001 M EDTA in ddH2O) and incubated on ice for 5 min, then filtered through 100 µm cell strainer and washed with PBS.<br><br>For isolation of milk cells, milk was diluted in a 1:1 ratio with PBS and centrifuged at 800g for 20 minutes at 15°C. The lipid layer and skim milk were removed, and the cell pellet was washed twice in PBS by centrifugation at 400g for 5 minutes and resuspension in PBS.<br><br>For histology, mice were euthanized through CO2 asphyxiation and perfused with PBS. Mammary fat pads were removed, fixed in 4% PFA (Morphisto) for 6-24 hours at 4°C, washed in PBS followed by incubation in 30% sucrose in PBS at 4°C for 1 to 5 days. The tissue was then embedded in Cryo Embedding Medium (Medite) and frozen on dry ice.
Instrument	Flow cytometry analysis was performed on LSR II Fortessa, BD FACSymphony and Cytek Aurora. Samples for scRNAseq were sorted on Aria III and S6 cell sorters. Sequencing was performed on NovaSeq6000 platform. Imaging was performed on Leica SP8 Falcon and Leica SP5 microscopes.
Software	FlowJo 10.6.2 and 10.8.1 (Tree star), BD FACS DIVA and SpectroFlo® Software, Seurat v3 and v4, LAS X, Imaris
Cell population abundance	Frequencies and cell counts per mammary gland are specified in the figure legends.
Gating strategy	In general, cells were gated based on FSC-A and SSC-A to exclude debris, doublets were excluded by FSC-Area vs. FSC-Height gating. Dead cells were excluded from the analysis using Zombie NIR fixable staining reagent (BioLegend). Supplemental gating strategies are provided in Extended Data.

☒ Tick this box to confirm that a figure exemplifying the gating strategy is provided in the Supplementary Information.
